# Isometric hand grip strength measured by the Nintendo Wii Balance Board – a reliable new method

**DOI:** 10.1186/s12891-016-0907-0

**Published:** 2016-02-03

**Authors:** A. W. Blomkvist, S. Andersen, E. D. de Bruin, M. G. Jorgensen

**Affiliations:** Department of Geriatric and Internal Medicine, Aalborg University Hospital, Aalborg, Denmark; Department of Clinical Medicine, Aalborg University, Aalborg, Denmark; Department of Health Sciences and Technology, Institute of Human Movement Sciences and Sport, ETH Zurich, Zurich, Switzerland

**Keywords:** Nintendo Wii Balance Board, Isometric hand grip strength, Reliability, Reproducibility, Test-retest, Validity, Jamar hand dynamometer

## Abstract

**Background:**

Low hand grip strength is a strong predictor for both long-term and short-term disability and mortality. The Nintendo Wii Balance Board (WBB) is an inexpensive, portable, wide-spread instrument with the potential for multiple purposes in assessing clinically relevant measures including muscle strength. The purpose of the study was to explore intrarater reliability and concurrent validity of the WBB by comparing it to the Jamar hand dynamometer.

**Method:**

Intra-rater test-retest cohort design with randomized validity testing on the first session. Using custom WBB software, thirty old adults (69.0 ± 4.2 years of age) were studied for reproducibility and concurrent validity compared to the Jamar hand dynamometer. Reproducibility was tested for dominant and non-dominant hands during the same time-of-day, one week apart. Intraclass correlation coefficient (ICC) and standard error of measurement (SEM) and limits of agreement (LOA) were calculated to describe relative and absolute reproducibility respectively. To describe concurrent validity, Pearson’s product–moment correlation and ICC was calculated.

**Results:**

Reproducibility was high with ICC values of >0.948 across all measures. Both SEM and LOA were low (0.2-0.5 kg and 2.7-4.2 kg, respectively) in both the dominant and non-dominant hand. For validity, Pearson correlations were high (0.80-0.88) and ICC values were fair to good (0.763-0.803).

**Conclusion:**

Reproducibility for WBB was high for relative measures and acceptable for absolute measures. In addition, concurrent validity between the Jamar hand dynamometer and the WBB was acceptable. Thus, the WBB may be a valid instrument to assess hand grip strength in older adults.

## Background

Muscle function is pivotal to overall physical fitness and a change in muscle strength is an important risk factor for functional decline, independent of disease processes [1]. Hence, the assessment of muscle function is an important measure in many situations. It may be assessed by proxies such as muscle mass or muscle strength. Compared to muscle mass, it is easier and more reliable to measure muscle function in terms of strength, particularly hand grip strength (HGS) [2, 3]. Accordingly, HGS is a strong predictor of future disability [4] and mortality [2, 3, 5–7] in the old and middle-aged [4, 7, 8]. In addition to being a marker for nutritional status [9, 10], low HGS is also associated with increased risk of postoperative complications, extended hospitalisation, higher re-submission rates and increased short-term mortality following acute admission [9–11].

In clinical settings, there are several methods for assessing muscle strength. Manual muscle testing (using a subjective scale from 0 to 5) is one of the most commonly used methods because of its simplicity and speed, however, a serious drawback is its crudeness [12, 13]. Another way of evaluating HGS is by using an objective handgrip dynamometer, which can be hydraulic, pneumatic, mechanical or electrical. The handgrip dynamometer has shown high reliability and validity when appropriately calibrated [14–16] and it can be useful for identifying individuals at high risk of poor disease outcomes [17]. The gold standard by which other dynamometers are evaluated is the Jamar hand dynamometer (JD) [16].

Most handgrip dynamometers are primarily found in the hands of health care professionals as they only measure HGS and prices range from 250 to 1484 USD. In contrast, the Nintendo Wii Balance Board (WBB) is widely available globally and it sells at approximately 60 USD. Moreover, the WBB has been demonstrated to be a reliable and valid instrument for the assessment of other physical characteristics such as balance [18] and reaction-time [19]. In 2014, an American research group demonstrated that both new and used WBB recorded static forces accurately in a laboratory setting [20]. Inspired by these findings, researchers at Aalborg University Hospital have developed software that enabled isometric strength recordings to be performed using the WBB. This software has shown high reproducibility and concurrent validity for measuring isometric muscle strength in the lower limbs [21]. Next, we want to establish whether this software can be used for isometric HGS testing. Hence, the aim of this study was (1) to explore both relative and absolute reproducibility of the WBB to measure HGS in the dominate and the non-dominate hand and (2) to explore concurrent validity when compared to the gold standard, the JD.

## Method

### Design

Following the guidelines for reporting reliability and agreement studies (GRRAS) [22], we tested the intra-rater reproducibility of the WBB performing tests one week apart. Concurrent validity was also explored by comparing the WBB to JD on the first session. Participants were randomized to start with either the WBB or JD in order to avoid order effects.

### Study-population

Thirty older adults were recruited using the member lists from senior citizen clubs and organizations in Aalborg, Denmark. Using telephone interview, participants were included if they were 65 years or more, willing and able to come to the hospital twice within a week by themselves, and able to pass a small custom dementia screening (correctly answering the current year, month and prime minister of Denmark). Participants were excluded if they had acute illness within the previous 3 weeks, orthopaedic surgery on upper or lower limbs within 6 months or neurologic disease (e.g. Parkinson’s disease, severe dementia). All participants gave written consent and the study was approved by the regional ethics committee, The North Denmark Region Committee on Health Research Ethics, which is appointed by The Regional Council of the North Denmark Region.

### Overall experimental and calibration procedures

Participant characteristics such as height, weight, handedness, number of drugs taken and physical activity in hours per week were obtained prior to testing. All tests were performed at the same time-of-day, in the same clinical examination room at Aalborg University Hospital and by the same rater. The rater was a trained physiologist (MGJ). Devices were calibrated by applying known weights of 0.2, 0.5, 1.0, 1.5, 2, 4, 5, 8, 10, 20, 30 and 50 kg to the force transducers.

### WBB

WBB is a rigid square-shaped platform with four uni-axial vertial stain gauge transducers in the corners. Using Bluetooth HID wireless and custom programs written in C#, data was streamed to a computer (Lenovo Yoga Pro, Windows 8). The software recorded the isometric force-time curve from the sensor values reported as four channels of 16-bit digital data samples at approximately 100 Hz and subsequently filtered using a 4th order Butterworth filter (cut-off frequency 20 Hz). The resulting accuracy of the software is 100 gram on the whole measurement range (from 0 to 300 kg).

**Fig. 1:**
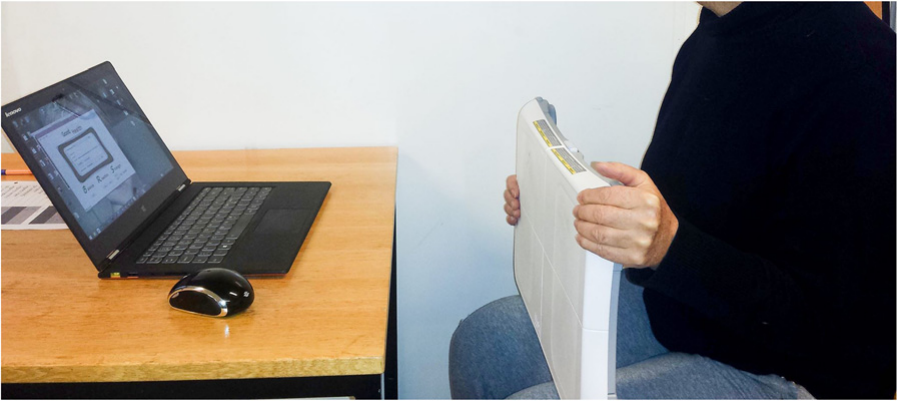
Demonstration of a left hand grip strength measurement by pressing the upper left corner of the Nintendo Wii balance board

Before starting the actual tests, participants received a set of standard instructions and demonstration of the procedure. Afterwards they were seated in a standard chair (seating height 43 cm), which was used for all tests. Participants were then asked to hold the WBB with their left and right hand around the middle of the WBB with the lower face of the board towards their torso. All tests were initiated with the right hand, which held and squeezed the upper right corner. This was followed by the left hand holding and squeezing the upper left corner, as illustrated in Fig. 1. Prior to the actual testing, 2–3 sub-maximal recordings were performed. This served both as habituation and warm-up. After the warm-up, the actual tests were performed with a total of three measurements per hand alternating between right and left hand. The participants were encouraged to squeeze as long and as tightly as possible until a plateau had been reached. This took about 3–5 seconds and was visualized on the monitor which both the examiner and participant could see. The examiner instructed the participant when to stop. The participants rested their hands for 15 seconds before the next measurement.

### JD

The JD (Lafayette Instruments Company, USA) is the most widely cited dynamometer in the literature and it is accepted as the gold standard by which other dynamometers are evaluated. It reads force in both kilograms and pounds, with markings at intervals of 2 kg. According to our calibration procedures, the JD required a minimum of 2 kg to make the manometer move, which may be inappropriate when measuring very weak patients. Accordingly, it has been reported that the measurement error of the JD is greater at lower loadings [23].

**Fig. 2:**
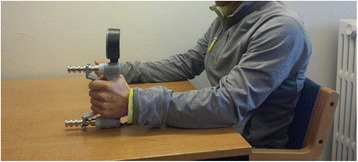
Demonstration of a left hand grip strength measurement by pressing the Jamar handdynamometer

Similar to the WBB test, participants received a set of standard instructions for the procedure followed by a demonstration. They performed 2–3 sub-maximal recordings prior to actual testing. In addition, participants rested their arm on a standard table (height 71 cm) with the JD initially in the right hand followed by the left hand. The hand was positioned with the thumb on one side of the handle, while the other fingers were on the other side (see Fig. 2). The handle was set to position no 2. Similar to the WBB, a total of three measurements per hand were completed and the participants were encouraged to squeeze as long and tight as possible until a plateau had been reached.

### Statistics

Data are presented as mean ± standard deviation (SD), and all statistical analyses were performed using SPSS (version 22). The dominant and non-dominant hands were analysed separately and measurements were presented as first measurement, mean of two measurements, mean of three measurements and highest value out of three measurements. For reproducibility, the difference between session one and two (for WBB) was tested for normal distribution both statistically (Shapiro-Wilk) and visually (histogram). Further, the difference between each participant’s individual score from the mean of the measurements in both sessions was plotted in a simple scatter plot for signs of heteroscedasticity [24]. Paired *t* test was used to explore systematic bias between sessions. For relative reproducibility, intra-class correlation coefficient (ICC) was calculated with a 95 % confidence intervals [25] using absolute agreement in a two-way mixed model, and the results of a single measurement was reported. The ICCs were interpreted based on the recommended ranges of poor (<0.69), fair (0.70-0.79), good (0.80-0.89), and high (0.90-1.00) for both relative reproducibility and validity [26]. For absolute reproducibility, the standard error of measurement (SEM) and limits of agreement (LOA) was calculated using the SD of the participants difference score between the two session multiplied by $\sqrt{1-ICC}$ and 1.96, respectively [27]. The absolute values were also presented as percentages by dividing SEM and LOA with the mean value of all participants for both sessions.

For validity, Pearson’s product–moment correlation between WBB and JD from session one were calculated for the first measurement, mean of two measurements, mean of three measurements and highest value of all three measurements. The correlations were interpreted as high (>0.70), moderate (0.50-0.69), low (0.26-0.49), and absent (0.00-0.25) [26]. To further support the validity analysis we included a calculation of ICC using a two-way mixed consistency model and reporting results of a single measurement.

## Results

The study-population consisted of 18 women and 12 men with a mean age of 69 ± 4.2 years. Characteristics included height 168.5 ± 6.9 cm, weight 72.5 ± 13.7 kg, BMI 25.5 ± 4.2 kg/m^2^, number of medications 1.5 ± 1.7, while physical activity was 8.1 ± 3.5 hours per week. Two participants did not show up for session two. Their results were excluded for the reproducibility analysis, while their measurements from the first session were retained for the validity analysis.

**Fig. 3:**
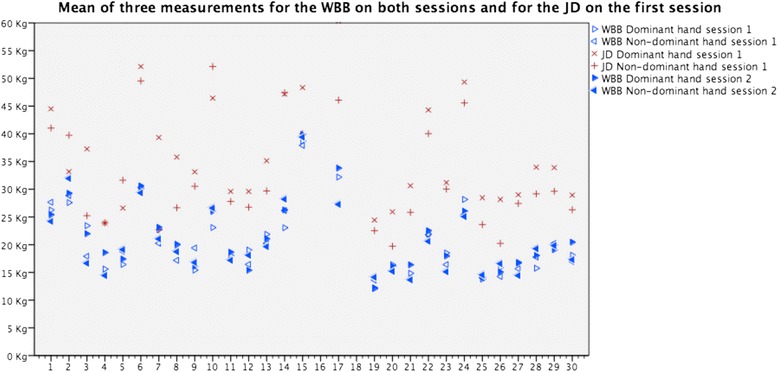
Mean of three measurements for the WBB on both sessions and for the JD. Vertical axis shows the results in kilogram. Horizontal axis represents each participant. Results from two participants, who did not show up for second session, are omitted (no 16 and 18)

In Fig. 3, the mean value for three measurements is shown for the WBB on both sessions and the JD. The between-subjects variation is greater than the within-subject variation. Also, the JD reads on average higher values than the WBB. Post hoc analysis using the mean score of three measurements from the first session demonstrates an average difference of 15.4 ± 5.5 kg for the dominant hand and 11.9 ± 5.5 kg for the non-dominant hand with the JD giving higher values.

Reproducibility results for dominant and non-dominant hands are shown in Tables 1 and 2, respectively. ICC values were 0.948-0.976, SEM between 0.2 and 0.5 kg and LOA were between 2.7 and 4.2 kg across all measurements. There were no statistically significant differences between sessions one and two, and there were no visual signs of heteroscedasticity.

**Table 1:** Results from the reproducibility analysis for the dominant hand

Dominant hand	Session 1		Session 2					
Measurement(s)	MEAN	SD	MEAN	SD	M-Diff (paired T-test)	ICC [95 % CI]	SEM (SEM%)	LOA (LOA%)
First measurement	21.74	1.19	21.94	1.29	0.20 (n.s.)	0.955 [.906-.979]	0.4 (1.9)	3.8 (17.6)
Mean of the two first measurements	21.54	1.19	21.70	1.27	0.16 (n.s.)	0.970 [.937-.986]	0.3 (1.3)	3.1 (14.3)
Mean of all three measurements	21.37	1.18	21.60	1.23	0.23 (n.s.)	0.966 [.927-.985]	0.3 (1.4)	3.1 (14.6)
Highest value of all three measurements	22.33	1.19	22.81	1.34	0.48 (n.s.)	0.960 [.905-.982]	0.4 (1.6)	3.4 (15.4)

Mean, standard deviation (SD), standard error of measurement (SEM) and limits of agreement (LOA) in kilograms. M-diff is the mean difference in kilograms between the mean of the two sessions and the comparison using the paired T-test. n.s. not significant (all p-values are less than 0.001). SEM and LOA are also given in percentages (SEM% and LOA%). Intraclass correlation coefficient (ICC) with 95 % confidence intervals [95 % CI] are given. Results are given for one measurement, mean of two measurements, mean of three measurements and highest value of three measurements

**Table 2:** Results from the reproducibility analysis for the non-dominant hand

Non-dominant hand	Session 1		Session 2					
Measurement(s)	MEAN	SD	MEAN	SD	M-Diff (paired T-test)	ICC [95 % CI]	SEM (SEM%)	LOA (LOA%)
First measurement	21.47	1.28	20.78	1.27	−0.69 (n.s.)	0.948 [.891-.975]	0.5 (2.4)	4.2 (20.1)
Mean of the two first measurements	21.08	1.22	20.68	1.25	−0.40 (n.s.)	0.973 [.943-.988]	0.2 (1.2)	2.9 (14.3)
Mean of all three measurements	21.30	1.20	20.65	1.23	−0.65 (n.s.)	0.976 [.947-.989]	0.2 (1.1)	2.7 (13.3)
Highest value of all three measurements	22.30	1.24	21.61	1.32	−0.69 (n.s.)	0.961 [.917-.982]	0.4 (1.7)	3.7 (16.8)

Mean, standard deviation (SD), standard error of measurement (SEM) and limits of agreement (LOA) in kilograms. M-diff is the mean difference in kilograms between the mean of the two sessions and the comparison using the paired T-test. n.s. not significant (all p-values are less than 0.001). SEM and LOA are also given in percentages (SEM% and LOA%). Intraclass correlation coefficient (ICC) with 95 % confidence intervals [95 % CI] are given. Results are given for one measurement, mean of two measurements, mean of three measurements and highest value of three measurements

Validity results are shown in Tables 3 and 4. The Pearson correlations between WBB and JD for all measurements were between 0.80 and 0.88 with the differences being statistically significant, and ICC values were between 0.793 and 0.803.

**Table 3:** Results from the concurrent validity analysis for the dominant hand

Dominant hand		
Measurement(s)	Pearson’s product–moment correlation	ICC [95 % CI]
First measurement	0.88 (*p* < 0.001)	.793 [.610-.896]
Mean of the two first measurements	0.88 (*p* < 0.001)	.803 [.626-.901]
Mean of all three measurements	0.86 (*p* < 0.001)	.786 [.597-.892]
Highest value of all three measurements	0.87 (*p* < 0.001)	.791 [.606-.895]

Intraclass correlation coefficient (ICC) with 95 % confidence intervals [95 % CI] and Pearson’s product–moment correlation with p-values are given for one measurement, mean of two measurements, mean of three measurements and highest value of three measurements. All measurements are from session 1

**Table 4:** Results from the concurrent validity analysis for the non-dominant hand

Non-dominant hand		
Measurement(s)	Pearson’s product–moment correlation	ICC [95 % CI]
First measurement	0.80 (*p* < 0.001)	.763 [.559-.880]
Mean of the two first measurements	0.80 (*p* < 0.001)	.748 [.535-.872]
Mean of all three measurements	0.82 (*p* < 0.001)	.768 [.562-.882]
Highest value of all three measurements	0.86 (*p* < 0.001)	.794 [.611-.896]

Intraclass correlation coefficient (ICC) with 95 % confidence intervals [95 % CI] and Pearson’s product–moment correlation with p-values are given for one measurement, mean of two measurements, mean of three measurements and highest value of three measurements. All measurements are from session 1

## Discussion

To the best of our knowledge, this is the first report measuring HGS using the WBB. The results demonstrated good reproducibility for the WBB in measuring isometric HGS with ICC values similar to JD [23, 28–33], the gold standard for measuring HGS. In addition, SEM and LOA values were comparable to or lower than [34] those observed with the JD, reflecting an acceptable absolute reproducibility. Moreover, we found a high concurrent validity between the JD and WBB with Pearson’s product–moment correlation averaging 0.85. As expected, the Pearson correlation was somewhat lower than reported correlations between the JD and other handgrip dynamometers [35, 36]. Still, the correlation was higher than that found between other HGS measurement techniques, such as the sphygmomanometer [37], grip-ball [32] and vigorimeter [38], and comparable to stationary alternatives, such as the BTW work simulator [39].

On the other hand, the ICC values between the JD and WBB were a bit weaker than the Pearson’s correlations, ranging between fair and good (0.793-0.803). The confidence intervals for the ICC validity analysis were significantly wider than for the reproducibility analysis, spanning from 0.525 to 0.901 and thus encompassing the full range from poor to high correlations. Hence, we have less confidence on the ICCs for the validity analysis, which must be interpreted with caution. The result from our study also indicate lower errors of measurement when considering the mean value of two or three measurements rather than one measurement or the maximum of three measurements, which is consistent with other studies [40].

Although the WBB showed a acceptable correlation with the JD, there was a systematic difference in the results. On average this was 15.4 ± 5.5 kg for the dominant hand and 11.9 ± 5.5 kg for the non-dominant hand with the JD giving higher values. Hence, there is an inter-instrument difference between the WBB and the JD, and the instruments are not interchangeable. However, this lack of agreement has also been found between different dynamometers [35, 41, 42] and even between different models of JD [43]. These studies [35, 41, 42] are consistent with our results in that the JD tends to give higher values when compared to other instruments.

Comparing our results from the dominant and non-dominant hand there is similar reproducibility between hands, but a somewhat better correlation with the JD for the dominant side, both with Pearson’s correlation (0.87 vs 0.82 on average) and ICC (.793 vs .768). The cause for this difference cannot be deduced from our results, but it might be expected that the strength difference between the two sides will result in lower correlation for the weaker side, i.e. non-dominant side (about 5 % weaker according to our results), since the JD allows assessment to the nearest kilogram, while the WBB allows assessment down to the nearest 100 gram. In combination with the increased measurement error for lower loadings with the JD, this may explain the difference observed.

One limitation with the WBB method for measuring HGS is the lack of adjustable handles to accommodate different hand sizes. Thus, the effect of hand size on our method is unknown. Still, this is the first investigation of HGS using the WBB. Compared to the above mentioned techniques the WBB has advantages in that it is a low-cost, portable and wide-spread tool. Furthermore, it has the potential for multiple roles in the clinical setting. This may include objectified measurements of reaction-time [19], balance [18] and lower limb muscle strength [21]. The data presented here demonstrate the applicability of an additional facility, the HGS. The WBB has also been successfully used as an intervention tool for balance in healthy eldery [44], as well as in chronic diseases [45, 46] and for physical rehabilitation [47–49]. Finally, the multiple use of one instrument with rapid and automatic transferral of measurements to the computer system prevents loss of results and error in reporting, and it supports optimal use of staff time.

This study has both strengths and weaknesses. Firstly, we did not investigate inter-rater reliability. The impact on WBB results is likely to be limited as the results are read automatically and hand positioning is likely to be similar between raters. Secondly, the participants positioning deviates from the standardised procedure recommended by American Society of Hand Therapists [16], but only by having a slightly more extended elbow position. The magnitude of this limitation is likely to be minimal. Thirdly, we only investigated independent older adults with a high level of functioning, as evaluated by their physical activity. Caution should be taken for generalizing the results to other age groups and populations. Still, this study had a sufficient number of participants for the purpose under study, and the methods have been reported in sufficient detail with the relevant statistics according to the GRRAS.

## Conclusion

Reproducibility for WBB was high for relative measures and acceptable for absolute measures in both dominant and non-dominant hands in a cohort of older adults. In addition, an acceptable concurrent validity was found between the JD and the WBB. Thus, the WBB appears to be a valid and reliable instrument when assessing HGS in older adults and may be a useful tool in clinical settings. Further research should aim to establish the inter-rater reproducibility and to explore the method in other populations.

### Availability of Data and Materials

Raw material can be provided upon request, please contact AWB or MGJ.

## Abbreviations

HGS: Hand grip strength
WBB: Nintendo Wii Balance Board
JD: Jamar handdynamometer
SEM: Standard error of measurement
LOA: Limits of agreement
ICC: Intraclass correlation coefficient
GRRAS: Guidelines for reporting reliability and agreement studies
